# The Investigation of the Cardiovascular and Sudomotor Autonomic Nervous System—A Review

**DOI:** 10.3389/fneur.2019.00053

**Published:** 2019-02-12

**Authors:** Tjalf Ziemssen, Timo Siepmann

**Affiliations:** ^1^Autonomic and Neuroendocrinological Functional Laboratory, Center of Clinical Neuroscience, Neurological University Clinic Carl Gustav Carus, Dresden, Germany; ^2^Neurological University Clinic Carl Gustav Carus, Dresden, Germany

**Keywords:** autonomic nervous system, laboratory evaluation of cardiovascular function, orthostatic tests, valsalva maneuver, heart rate variability, axon reflex, sympathetic, sudomotor function

## Abstract

The autonomic nervous system as operating system of the human organism permeats all organ systems with its pathways permeating that it is involved with virtually all diseases. Anatomically a central part, an afferent part and sympathetic and parasympathetic efferent system can be distinguished. Among the different functional subsystems of the autonomic nervous system, the cardiovascular autonomic nervous system is most frequently examined with easily recordable cardiovascular biosignals as heart rate and blood pressure. Although less widely established, sudomotor tests pose a useful supplement to cardiovascular autonomic assessment as impaired neurogenic sweating belongs to the earliest clinical signs of various autonomic neuropathies as well as neurodegenerative disorders and significantly reduces quality of life. Clinically at first, the autonomic nervous system is assessed with a detailed history of clinical autonomic function and a general clinical examination. As a lof of confounding factors can influence autonomic testing, subjects should be adequately prepared in a standardized way. Autonomic testing is usually performed in that way that the response of the autonomic nervous system to a well-defined challenge is recorded. As no single cardiovascular autonomic test is sufficiently reliable, it is recommended to use a combination of different approaches, an autonomic test battery including test to measure parasympathetic and sympathetic cardiovascular function (deep breathing test, Valsalva maneuver, tilt, or pressor test). More specialized tests include carotid sinus massage, assessment of baroreceptor reflex function, pharmacological tests or cardiac, and regional hemodynamic measurements. Techniques to measure functional integrity of sudomotor nerves include the quantitative sudomotor axon reflex sweat test, analysis of the sympathetic skin response as well as the thermoregulatory sweat test. In addition to these rather established techniques more recent developments have been introduced to reduce technical demands and interindividual variability such as the quantitative direct and indirect axon reflex testing or sudoscan. However, diagnostic accuracy of these tests remains to be determined. We reviewed the current literature on currently available autonomic cardiovascular and sudomotor tests with a focus on their physiological and technical mechanisms as well as their diagnostic value in the scientific and clinical setting.

## Introduction

Functional disorders in the autonomic or vegetative nervous system play an extremely important role in the suffering spectrum of many patients and in everyday medical practice (1). There is absolutely no disease or ailment that does not involve autonomic regulation or innervation disorders. The importance of the autonomic nervous system lies, among other things, in the fact that every organ of the human body is innervated and thus regulated by the autonomic nervous system (2, 3). Thus, the autonomic nervous system tries to restore “sympathy” (Galen) between the individual functional systems after a disturbance of the balance of the human body with the help of certain adjustment reactions. The cardiovascular system is most frequently examined in autonomic functional diagnostics (4). On the one hand, the measurement parameters of interest such as heart rate or blood pressure can be measured relatively easily and on the other hand prognostic statements can be made for patients e.g., after myocardial infarction or with diabetic neuropathy (5).

### Anatomical Basics

Different areas of the brain are regarded as components of a complex central autonomic network that processes incoming information from the periphery (**autonomic afferences**) and generates a corresponding stimulus response to the peripheral target organs (**autonomic efference**) (6). Within this efferent system, two mostly opposing components are traditionally distinguished (2, 3):

- The sympathetic nervous system is the so-called “emergency system.” After activation, it leads, among other things, to pupil dilation, acceleration of the heart rate, increase in heart power, and vascular resistance. After the sympathetic nerves have left the spinal cord in the thoracic and lumbar vertebrae, they still have to be switched over to the second sympathetic neuron either in prevertebral or paravertebral ganglia. If there is a disturbance prior to this ganglion switching, it is called preganglionic damage, otherwise it is called postganglionic damage. Acetylcholine is released as a transmitter at all preganglionic nerve endings and postganglionic at the sweat glands, while norepinephrine is released postganglionically at the effector organs with the exception of the sweat glands.
- The parasympathetic nervous system is simply understood as an opponent of the sympathetic system, i.e., as a “resting or recovery system” which, for example, plays a major role in controlling digestion. After activation, it leads, among other things, to pupil reduction, decrease in heart rate and activation of digestion. In the upper part it also supplies the eyes, tear and salivary glands, heart, lungs as well as the digestive tract after ganglion switching. The nerve fibers emerging from the coccyx are crucial in controlling the urinary tract and the lower digestive tract. The primary neurotransmitter of postganglionic parasympathetic neurons is acetylcholine.

Most organs of the body are innervated by efferent autonomic nerve fibers from both the sympathetic and parasympathetic nervous system. The cholinergic cardiac innervation delivers substantial supply to the ventricles in particular. Cholinergic/noradrenergic co-transmission is apparently a unique feature of the primate autonomic sympathetic nervous system (7). However, over the recent decades we learned that the autonomic nervous system does not operate only with acetylcholine and noradrenaline as classical transmitters (8), A growing number of different especially peptidergic signaling molecules (e.g., VIP, PACAP, CGRP, Substance P) have been described (9). This neuropeptide co-transmission in the autonomic nervous system increases the flexibility to synapses and circuits, including the surprising range of degrees of freedom.

### Physiological Basics

In the regulation of the cardiovascular system, a sufficient blood supply to the tissue corresponding to the need must be ensured at all times as a decisive parameter. A sufficiently high blood pressure is essential for an adequate blood supply to the tissue. The autonomic nervous system with the **baroreceptor reflex** is decisively involved in its short-term regulation in particular (10, 11):

Special sensors in the area of the carotid sinus and the aortic arch can signal a drop in blood pressure via the cranial nerves IX and X to autonomic control centers in the brain, e.g., as a result of a change of position into the vertical body position (12). Certain circulatory centers in the brain stem, such as the nucleus tractus solitarii, process the signals and activate the sympathetic efference, which leads to an increased heart rate and inotropy, a mobilization of blood reserves from the venous system and an increase in systemic vascular resistance (13). At the same time, parasympathetic activity to the heart is reduced, which also contributes to an increase in heart rate.

The example of the baroreceptor reflex shows a basic principle of the autonomic nervous system, the **autonomic reflex arc** as a control loop (14, 15). Each of the various autonomic reflex arcs consists of an afferent, a central processing and an efferent component (16). The afferent signal mostly comes from specialized sensors such as the baroreceptor, which can detect changes in a biosignal and convert them into nerve impulses. The signal reaches the central nervous system via peripheral nerves or cranial nerves. After repeated neuronal switching, the afferent signal is further processed there in comparison with other signals and control information in higher regulation centers, whereby several specific central nervous processing centers usually exist for each reflex arc. From there, an efferent response is generated to the respective specific effector organs of the control loop, e.g., the smooth vascular muscles. The reaction of the effector organ helps to eliminate the disturbed state previously detected by the sensors with the help of a specific counter-regulatory mechanism.

The sudomotor autonomic system complements cardiovascular autonomic control in upholding a stable thermoregulation of the human body. The two major mechanisms involved in maintaining a constant body temperature of 37°C are dilation/constriction of cutaneous vessels and sweat production. The cerebral center of thermoregulation and sudomotor function is the hypothalamus which processes input from visceral and peripheral thermoreceptors to determine sudomotor activity via two separate efferent pathways to regulate temperature control. These pathways are somatic motor fibers mediating an increase in body temperature by inducing muscle shivering as well as sympathetic fibers regulating blood vessel and sudomotor function, the latter resulting in a decrease of body temperature upon activation (17). Efferent sympathetic sudomotor pathways originate from the hypothalamus and travel via the pons and the lateral reticular medulla to the intermediolateral column. After leaving the spinal cord, the preganglionic cholinergic neurons of the intermediolateral column form synapses with postganglionic sympathetic cholinergic sudomotor neurons. Postganglionic control of cutaneous sweat glands is mediated by axons of these neurons which innervate the skin as unmyelinated C-fibers. Up to 3.5 liters of sweat can be produced per day depending on thermoregulatory demands. Autonomic control of sweat production is in large parts influenced by environmental factors, such as humidity, temperature and is additionally dependent of age and gender. Due to high complexity and susceptibility toward environmental factors as well as inter and intra subject variability of sweat gland density sudomotor function assessment comprises some of the technically most demanding tests of the autonomic nervous system.

### Anamnesis of the Cardiovascular Autonomic Nervous System

Especially in patients with a suspected disorder of the cardiovascular autonomic nervous system, a good symptom as well as system-oriented anamnesis and a clinical examination must be carried out before a possible visit to the autonomic function laboratory (Table 1) (18). A good anamnesis and clinical examination are usually much more effective than a “blind” functional diagnosis in an autonomic functional laboratory.

**Table 1:** Selection of important symptoms in anamnesis and examination of the cardiovascular autonomic nervous system.

**History of the cardiovascular system**	
•Blood pressure:	Orthostatic hypotension or syncopes—severity, frequency, duration
	• Triggers: Orthostasis, food intake, heat, physical exertion, etc.
	• Accompanying symptoms: anxiety, nausea, vomiting, dizziness, tiredness, tachycardia, disturbed vision or hearing, headache/neck/back pain, etc.
	• Subjective countermeasures: e.g., sitting down, crouching down, lying down
	Elevated blood pressure when lying down
•Heart rate:	Tachycardia at rest, during exercise or orthostasis, cardiac arrhythmias: disturbed sinus arrhythmia, non-variable heart rate
• Vasomotion:	heat/cold intolerance, sensitivity to cold (“cold acra”), skin trophics, skin color

### Indication for Autonomic Testing

Cardiovascular and sudomotor autonomic testing are indicated for a number of disorders and conditions. Clinically autonomic testing is useful in defining the presence of autonomic dysfunction, to provide differential diagnostic information, or to quantify autonomic function regarding their natural history and response to treatment (19). Main clinical indications are:

- When generalized autonomic failure is suspected (20). Generalized autonomic failure can be due to multiple system atrophy (MSA), pure autonomic failure (PAF) or autonomic neuropathies (diabetic, amyloid, Sjogren's syndrome, subacute autoimmune). Getting the diagnosis of this disorder is important to evaluate the prognosis.
- To detect limited autonomic failure, which can masquerade under a number of guises as chronic idiopathic anhidrosis, syncope, orthostatic intolerance or distal small fiber neuropathy.
- To differentiate benign autonomic disorders that can mimic life-threatening disorders. For instance, benign neurocardiogenic syncope need to be evaluated to rule out generalized autonomic failure. Chronic idiopathic anhidrosis can only be diagnosed with normal sympathetic and parasympathetic function.
- To evaluate orthostatic intolerance (21). Orthostatic intolerance including orthostatic hypotension, syncope or postural orthostatic tachycardia syndrome (POTS) means development of symptoms of cerebral hypoperfusion or autonomic overaction on orthostatic challenge with resolution on recumbency. Cardiovascular autonomic testing can evaluate the presence and severity of this condition and can differentiate whether underlying autonomic failure is present.
- To differentiate multiple system atrophy (MSA) from typical Parkinson's disease which can be done nicely by combining cardiovascular and sudomotor testing (22).
- To monitor the clinical course of autonomic dysfunction over time. The twin attributes of autonomic testing, quantitation and non-invasiveness, render it ideally suited to monitor the time course of autonomic dysfunction.
- To evaluate the response to treatment of autonomic dysfunction. The autonomic problems may lessen in response to treatment. When treatment is initiated, the quantitative methods of autonomic testing are needed to evaluate if the patient is responding in an adequate way.

## Cardiovascular Autonomic Functional Diagnostics in General

Functional diagnostics of the cardiovascular autonomic functional system is intended to help assess the functional integrity of certain autonomic reflex arcs in order to detect and localize disorders (23). To test such a reflex arc, the afferent pathway of the autonomic reflex arc must first be activated from the resting state of the human organism using a suitable stimulus such as an orthostatic test to examine the baroreceptor reflex. The test person should be in a relaxed state of rest, because otherwise the changes to be observed and evaluated cannot be understood as the effect of the disturbance stimulus used alone (24). Each test may therefore only be carried out after a sufficient rest period, also between the individual function tests. On the other side, the respective disturbance stimulus used must always be standardized in order to be able to determine the existence of a normal or pathological reaction to the disturbance stimulus by comparison with the cardiovascular stimulus response of a normal collective in individual cases (25). In order to assess the autonomic efferent reflex response, the nerve activity in the efferent autonomic nerve can be measured directly by microneurography (26). An indirect evaluation by measuring functional parameters of the effector organs, such as heart rate or blood pressure, is much easier and is therefore more common (4, 16).

The quality of a functional test of the cardiovascular autonomic nervous system depends on whether a conclusion can be made after the examination regarding the existence, type, severity, localization, prognosis of a dysfunction and the therapeutic effectiveness on the individual patient. The ideal autonomic functional test should be easy and safe to perform, clearly understandable, non-invasive, reproducible, sensitive and specific as well as suitable for long-term studies (6, 27).

Even though most autonomic functional tests are relatively easy to perform, the interpretation of the test results is often difficult due to the complexity of the individual reflex arcs and the fact that many external and internal disturbances can influence the test results (8). For example, age, physical fitness, patient medication or even room temperature in the examination room are important influencing factors in autonomic functional diagnostics. This requires strict standardization of patient preparation, test procedures and evaluation using standard values and test algorithms created for all laboratories (28). Therefore, Table 2 lists some recommendations for standardized patient preparation.

**Table 2:** Advice on patient preparation in cardiovascular autonomic function diagnostics.

**Important advice for patient preparation before autonomic testing**	
**48 h before the examination the following should be discontinued**
• Anticholinergics (e.g., antihistamines, antidepressants) • Sympathomimetics (α- and β-agonists) • Parasympathic mimetics • Mineralocorticoids (e.g., 9-α-Fludrocortisone) • Diuretics
**24 h before the examination the following should be discontinued**
• Sympathicolytics (α-antagonists, β-antagonists)
**12 h before the examination the following should be discontinued**
• Alcohol • Analgesics
**At the morning of the examination**
• No wearing of confining clothing • No corset • No support stockings
**3 h before the examination the following should be discontinued**
• Nicotine • coffee • food

## Biosignals Used in Cardiovascular Autonomic Function Diagnostics

The following biosignals are frequently used in cardiovascular autonomic function diagnostics:

- **Heart Rate:** Continuous ECG leads allow for a precise and current evaluation of the current heart rate. The heart rate, like blood pressure, is not a constant but a physiologically constantly changing biosignal. The investigation of this **heart rate variability (HRV)**, which is dependent on many factors (e.g., respiration), can provide information concerning the function of the cardiovascular autonomic nervous system (29). The days of manual evaluation of ECG strips are a thing of the past due to the automated and computer-aided algorithms available today. Although cardiac automaticity is intrinsically ensured by various pacemaker tissues, the autonomic nervous system regulates heart rate and rhythm in many ways. The variations of heart rate are modulated by a fine tuning of beat-to-beat control mechanisms by central (vasomotor and respiratory centers of the brain stem) and peripheral (oscillation of arterial blood pressure and respiration) oscillators (30). These oscillators generate rhythmic fluctuations of efferent nerve discharges which manifest themselves in short-term and long-term variations of the heart rate.
  An analysis of HRV allows for an evaluation of the status and function of the central oscillators, sympathetic and parasympathetic efference, humoral factors, and sinus node. The parasympathetic system mainly mediates reflective changes in heart rate to corresponding afferent signals of the arterial baroreceptors and the respiratory system, while the sympathetic system is mainly responsible for changes in heart rate to physical and mental stress. HRV is not clearly gender-dependent, but is clearly age-dependent (31). For more specific computer-aided HRV calculations, the ECG signal is sampled and digitized at a sampling frequency of 256 Hz (32). This is followed by an R-wave detection with subsequent RR interval calculation, whereby as many artifacts as possible are excluded by appropriate algorithms (27). Computer aided, the RR interval duration can be displayed over the interval number as a so-called tachogram.
  The raw data of the ECG recording must be statistically evaluated using appropriate methods due to the large amount of data. This can be done using the time domain analysis and the frequency domain analysis (33).
- The parameters of time domain analysis such as mean value, standard deviation or coefficient variation are relatively easy to calculate. Using mathematical statistical methods, essential and typical information can be filtered out of the measured signals and clearly displayed. Thus, the RR histogram shows the frequency of RR intervals at various lengths.
  In the frequency domain analysis, various spectral analytical methods allow conclusions to be drawn as to the variance distribution as a function of frequency and as to frequency-specific oscillations (33). Thus, not only the degree of variability described by the standard deviation can be determined, but also the corresponding oscillation frequency. Spectral analysis converts series of sequential RR intervals into a sum of sinusoidal functions of varying amplitudes and frequencies (34). The various spectral components are assigned to the different parts of the autonomic nervous system: The high frequency *HF (0.15–0.5 Hz) component* is mainly caused by efferent vagal activity. The low frequency *LF (0.05–0.15 Hz) component*, in contrast to the HF component, does not permit such an unambiguous identification (35). Thus, one can see a quantitative marker of efferent sympathetic activity with significant influences from parasympathetic activity in the LF component. The low frequency components [*very low frequency VLF (0.05 Hz) and ultralow frequency ULF]* cannot yet be adequately assessed in their results. The relationship between the low frequency (LF) and the high frequency component (HF) of spectral response is called sympathovagal balance by some authors. However, it should be noted that a change in the low frequency component (LF) can be mediated both sympathetically and parasympathetically, as already mentioned above (35).
- **Blood Pressure:** A major function of the sympathetic nervous system is the regulation of vascular tone (activity of the smooth vascular muscles), which in turn determines blood pressure and thus blood flow through the blood vessels. Like the heart rate, blood pressure also shows considerable variability (36).
  Blood pressure can be measured intermittently or continuously (37). Non-invasive, continuous blood pressure measurement is the gold standard for autonomic functional diagnostics and not only allows for the detection of short-term or slight blood pressure changes. It also allows for the quantitative assessment of important circulatory functions such as the baroreceptor reflex or blood pressure variability (10).
- **Respiration:** Although respiration has a decisive influence on functions of the autonomic nervous system such as heart rate variability, it is often not recorded or its influence neglected in the context of autonomic functional diagnostics (31). Recording respiration is important, for instance, in assessing whether the patient has a physiological respiratory pattern. For example, important diagnostic information for hyperventilation or apnea syndrome can be obtained from recording respiratory data. Secondly, knowledge of respiratory rate and volume is important for assessing respiration-dependent and respiration-independent oscillations in heart rate, blood pressure and blood flow. For example, slow, deep breaths can amplify the low frequency oscillations of heart rate and blood pressure to such an extent that this can lead to the false conclusion that increased sympathetic activity is the cause of this effect (38).
- **Blood flow:** Because vascular tone is not accessible for direct measurement, blood flow is used as the typical measurement for assessing vascular tone and thus the sympathetic vasomotor system (39). A quantitative assessment of blood flow requires the use of special methods based either on the principle of measuring changes in tissue temperature *(thermometry)*, the principle of volume change by blood flow *(plethysmography)* or the principle of the Doppler effect *(laser Doppler and Doppler sonography)*. It could be used to diagnose endothelial dysfunction (40).

## The Most Important Cardiovascular Autonomic Function Tests

### Deep Metronomic Breathing

#### Physiology

Physiologically, an increase in heart rate can be observed while inhaling and a decrease in heart rate while exhaling, which is mainly due to changing parasympathetic activity. The dependence of the heart rate on respiration is called respiratory sinus arrhythmia (RSA). During a metronomic respiration with six deep breaths per minute, maximum values of respiratory HRV are reached that can be compared to established norm values (28, 31). In addition to RSA, the predominant periodic fluctuations of the heart rate are the baroreceptor reflex heart rate change (so-called 10 s rhythm, and also known as the 3rd order blood pressure waves or described as Traube-Hering-Mayer waves) and the thermoregulatory heart rate change (41).

#### Implementation

In order to gain information about heart rate variability, the test person is instructed to breathe deeply and evenly so that the inhalation and exhalation phases each last 5 s. Avoid prolonging this test beyond 2 min because the hypocapnia that occurs will result in an increase in heart rate and reduced HRV.

#### Assessment

A simple assessment of HRV in metronomic respiration allows for the calculation of the strongly age-dependent quotient from the longest heart rate intervals during exhalation to the shortest intervals during inhalation **(I:E Ratio)** (42–45). For metronomic breathing at six breaths per minute, this age-dependent quotient should be >1.2. The E-I difference is the difference of the RR intervals during exhalation minus the RR intervals during inhalation during metronomic respiration. Physiological values are >15 beats per minute (bpm), threshold 11–14 bpm, pathological from 10 bpm.

### Orthostatic Maneuver

#### Physiology

The simplest and most commonly used method for assessing cardiovascular feedback is to measure cardiovascular parameters (such as heart rate, blood pressure, noradrenaline concentration) during a change from horizontal to vertical body position (46). During the transition from lying to standing, a displacement of 400–600 ml of blood into the leg veins occurs as a result of hydrostatic pressure changes (47). This leads to a short-term reduction in the venous return flow to the heart, which in turn reduces the heart beat volume and thus the arterial blood pressure. Due to the very rapid onset of compensatory mechanisms, arterial blood pressure changes only slightly in healthy patients. However, in 10–15% of all people, orthostatic circulatory disorders are observed due to insufficiency of these compensatory mechanisms (48).

#### Implementation

Orthostatic test can be carried out actively (Schellong test) by the test person standing up independently, or passively (tilt table test) using a tilting table (optimum tilting angle of 60°) (49). Both methods differ from each other especially in terms of their initial cardiovascular reactions (16). The blood pressure should be recorded continuously or intermittently (every 2.5 min) over an interval of 5–10 min before and at least 10 min after the change in position, parallel to a continuous ECG. During the test, patients must avoid both hyperventilation and a Valsalva maneuver.

#### Assessment

The heart rate and blood pressure changes recorded during the test are evaluated:

- Heart rate changes within the first 30 s after getting up allow for an assessment of the cardiac parasympathetic system. The ratio of the longest RR interval at about 30 heartbeats to the shortest RR interval at about 15 heartbeats is defined as “30:15 ratio” or “Ewing ratio” and represents a reproducible age-dependent index of cardiovagal function (39). Modern devices no longer calculate the pure 30:15 ratio, but the quotient of the longest RR interval between 20 and 40th heartbeat and the shortest RR interval between 5 and 25th heartbeat. Physiologically, the 30:15 ratio has a value >1.04.
- In orthostatic diagnostics, a systolic or diastolic blood pressure drop of at least 20 or 10 mmHg within 3 min after active placement or erection on the tilt table indicates so-called **orthostatic hypotension** (50). If the orthostatic symptoms are so severe that the patient cannot stand for at least 3 min, the standing time until the occurrence of orthostatic-dependent symptoms is recorded.

For checking syncope or suspected **postural tachycardia syndrome (POTS)**, an extended tilt test with a tilting time of up to 90 min can be performed. The main symptom of POTS is an excessive orthostatic tachycardia, which is at least 30 BpM/min higher than the initial frequency or persistently above 120 BpM/min within 10 min of changing position (51, 52). In a **neurocardiogenic syncope**, a sudden drop in blood pressure usually occurs only after a prolonged period of inactivity, usually without compensatory tachycardia, but with a bradycardia as well as presyncopal or syncopal symptoms (53).

Other special forms of the tilt table test are the orthostatic test after food intake (s*planchnic vasodilatation*) and the orthostatic test after physical exertion (*muscular vasodilatation*) as well as the tilt table test with negative pressure application in the area of the lower body half (*lower body negative pressure test*) by means of a special pressure chamber (54).

### Valsalva Maneuver

#### Physiology

The cardiovascular system's stimulus response to the Valsalva maneuver provides crucial information as to the integrity of the cardiovascular autonomic system e.g., the baroreceptor reflex. The hemodynamic response to a sudden, short-term increase in intrathoracic and abdominal pressure can be divided into four phases (55–57):

After a brief increase in blood pressure for 1–2 s (**Phase I**) due to mechanical compression of the aorta, arterial blood pressure decreases due to reduced cardiac preload and stroke volume (early **Phase II**). The decrease in systemic blood pressure is counteracted by an increase in heart rate and peripheral vasoconstriction, which causes arterial blood pressure to slowly rise back to a level at least equal to the previous blood pressure (late **Phase II**). After completion of the intrathoracic pressure increase, an excessive increase in diastolic and systolic blood pressure occurs after a brief mechanical drop in blood pressure (**Phase III**), because the venous reflux and thus the stroke volume suddenly increase, but the arterial vascular bed is constricted due to the still increased sympathetic activity (**Phase IV**). Due to the counter-regulatory activity of the baroreceptor reflex, the heart rate decreases.

#### Implementation

The patient generates the increase in intrathoracic and intraabdominal pressure of 40 mmHg for 10–20 s by exhaling through a special mouthpiece (e.g., a 5 or 10 ml syringe, the plunger rod of which has been removed and which is fitted with a small hole and connected to a blood pressure measuring device instead of a needle) (58). The deciding factor here is that the exhaling patient continuously generates a pressure between 20 and 40 mmHg with the epiglottis open (hence the hole in the syringe as a leak in the system). In order to be able to interpret the circulation reactions of the Valsalva maneuver more reliably, it is advisable to carry out the test three times. In general, the Valsalva maneuver can be considered both reproducible and sensitive. The position of the patient also plays an important role: the circulation effects are more pronounced in a seated position than when lying down. A conclusive test result depends to a large extent on the patient's cooperation.

#### Assessment

Continuous blood pressure recording is used to determine the changes in blood pressure caused by intrathoracic pressure changes, which allow the sympathetic activity and, if necessary, the severity of a functional disorder of the sympathetic cardiovascular system to be assessed (Table 3).

**Table 3:** Physiological and pathological changes in arterial blood pressure during the various phases of the Valsalva maneuver.

**Lesion**	**Phase I**	**Phase II (early)**	**Phase II (late)**	**Phase III**	**Phase IV**
None	Stress dependent increase in blood pressure	Arterial pressure drop	Increase in arterial blood pressure	Short-term drop in blood pressure	Excessive rise in blood pressure
Parasympathetic	Normal	Reduced blood pressure drop	Normal	Normal	Normal
Sympathetic, slight	Normal	Slight increase in blood pressure drop	Reduced to missing blood pressure increase	Normal	Slight reduction of the increase in blood pressure
Sympathetic, moderate	Normal	Significant increase in blood pressure drop	Missing blood pressure increase	Normal	Significant reduction in blood pressure increase
Sympathetic, severe	Normal	Severe drop in blood pressure	Missing blood pressure increase	Normal	Missing blood pressure increase

The heart rate changes during this maneuver are considered established sensitive and specific evaluation possibilities for the function of the parasympathetic functional system (25). The age-dependent Valsava ratio is calculated from the quotient of the longest RR interval (bradycardia) after the maneuver and the shortest RR interval (tachycardia) during or shortly after the end of the maneuver. Physiologically, a ratio is >1.21. The threshold range is between 1.11 and 1.20, pathological values are ≤ 1.10.

### Pressor Functional Tests

#### Physiology

The pressor stimuli applied here all lead to a stimulation of the sympathetic afference independent of the baroreceptor afference (45, 59). All pressor tests lead to an increase in blood pressure and heart rate. In the isometric hand grip test and cold pressor test, peripheral receptors are activated in addition to an important cerebral activation. The effect of other stimuli, such as the mental arithmetic test, depends primarily on cerebral activation.

#### Implementation

The **isometric hand grip test** evaluates changes in heart rate and blood pressure over a 3 min compression of a hand dynamometer or a partially inflated blood pressure cuff to approximately one third of the maximum fist closure force (60). In the **cold pressor test**, the stimulus lies in the 2 min immersion of one hand in ice water (61). In the **mental arithmetic test**, the patient must solve a complex sequential arithmetic problem during a 2 min interval (62).

#### Assessment

In most cases, the pressor function tests provide valuable information as to the function of the efferent sympathetic system, whereby isometric hand grip and cold pressor tests are the most conclusive, given that the overall examination time required is quite short. The increase in heart rate and blood pressure are assessed in relation to the respective stimulus. It should be noted that many factors, such as muscle weakness, can influence test results independently of the autonomic nervous system.

### Carotid Sinus Massage

#### Physiology

The carotid sinus massage examines the sensitivity of the baroreceptors of the carotid sinus and the parasympathetic efference. It should be used in syncope diagnosis if the patient's medical history suggests the suspicion of a hypersensitive carotid sinus. The maneuver leads physiologically to a moderate reduction in heart rate and possibly blood pressure as well.

#### Implementation

On a lying patient, the carotid sinus is observed with the head slightly bent backwards, palpated and a slight pressure is exerted for 20–30 s first on the right side and after a break of a few minutes also on the left side. If there is no cardiac response, the maneuver is repeated with increased massage pressure.

#### Assessment

Heart rate and blood pressure changes are assessed during sinus pressure application. While physiologically there is only a slight drop in heart rate and blood pressure, carotid sinus syndrome can cause asystole for more than 3 s and a significant drop in blood pressure (systolic blood pressure drop >50 mmHg) (19). Therefore, it may only be performed after strict diagnosis and Doppler sonographic examination of the cervical vessels as well as continuous ECG monitoring.

### Baroreceptor Sensitivity

The exact evaluation of the baroreceptor reflex, the so-called baroreceptor sensitivity, is becoming increasingly important for the diagnosis and understanding of pathophysiological relationships in numerous neurological and cardiological diseases due to the decisive role of this reflex for cardiovascular regulation as described above. In addition to the pharmacological examination (so-called Oxford method) (37), in which the effects of drug-induced blood pressure changes on the heart rate are assessed, the examination is also available by means of “neck suction,” i.e., a negative pressure stimulation of the neck region and thus of the baroreceptors, as well as the computer-assisted analysis of the relationships between spontaneous blood pressure and heart rate modulation. So trigonometric regressive spectral (TRS) analysis is a newly developed technique and solves several shortcomings of the traditional methods, mainly fast Fourier transform and autoregressive methods for spectral analysis (63). The analysis of a given electrocardiography recording (global data segment) is performed by multiple shifting local data segments, thus the software using TRS for spectral and baroreflex analysis is called multiple trigonometric regressive spectral analysis (MTRS) (27, 64).

### Special Functional Tests of the Autonomic Cardiovascular System

Another test described in the literature is the squatting test, in which squatting leads to an increase in systemic arterial pressure followed by bradycardia (24). Active rising again leads to a drop in arterial blood pressure, followed by tachycardia. The significance of this test is controversial.

Pharmacological methods help to check the sensitivity of different receptors and the functional integrity of the autonomic nervous system (65). For example, the response of the circulatory system to the application of noradrenaline allows the sensitivity of alpha-adrenoceptors activated by noradrenaline to be assessed. A physiological increase of the heart rate after administration of atropine indicates an intact cardiac vagal control. Tyramine releases noradrenaline from the granules and cytosol of postganglionic sympathetic neurons, so that their lesion lacks the characteristic increase in blood pressure and noradrenaline concentration after tyramine administration.

Pathological changes in metabolism and the function of neurotransmitters and hormones indicate the severity and localization of autonomic dysfunctions. The catecholamines—among them especially noradrenaline—are regarded as markers of sympathetic activity in plasma and urine, especially since there is a significant correlation between sympathetic nerve activity and noradrenaline concentration in plasma (66). During the orthostatic maneuver, blood samples can also be taken from patients before and after a change of position to determine neurotransmitters and hormones. However, it should be noted that the venous access is applied well before the start of the examination because the application of a venous access *per se* leads to significant changes in the hormone and neurotransmitter concentrations in the blood. In special cases, the determination of the hormones of the renin-angiotensin-aldosterone system or of vasopressin can also provide further information.

### Sudomotor Assessment: Clinical Significance

Sudomotor dysfunction can lead to either increased or decreased sweating, both of which can have severe implications to the subject's wellbeing and quality of life. Sudomotor dysfunction can occur both in central disorders affecting centers of sudomotor control such as acute ischemic stroke, multiple sclerosis and neurodegenerative syndromes as well as autonomic peripheral neuropathies which selectively affect unmyelinated and small, lightly myelinated nerve fibers (67, 68). Autonomic peripheral neuropathy is most frequently caused by diabetes but can also result from various diseases such as acute and chronic infections, primary or hereditary amyloidosis, paraneoplastic disorders such as Lambert–Eaton syndrome as well as some neurotoxins (e.g., cisplatin, vacor) (67). Patients suffering from sudomotor function may report either increased sweating in higher environmental temperatures or heat intolerance due to anhidrosis. These symptoms can significantly limit quality of life, for example, when patients with hyperhidrosis avoid social embarrassment due to visibly increased sweating in high temperature environments. Therefore, care of patients with sudomotor dysfunction requires detailed anamnesis of habits and social life. Furthermore, dyshidrosis related changes to epidermal moisturization may lead to hyperkeratosis, rhagades, ulcers or impaired wound healing highlighting the value of thorough visual inspection in these patients (17). According the concept of neurogenic inflammation, the dense innervation network of sensory and autonomic fibers in peripheral organ tissues can mediate a rapid local and systemic neurogenic modulation of immunity (69). So peripheral neurons can play a significant role in immune dysfunction in autoimmune and allergic diseases.

### Thermoregulatory Sweat Testing (TST)

The TST allows qualitative analysis of pre- and postganglionic sweating of the ventral body surface using quinizarin as color indicator to highlight sweating patters. Performing the TST requires a humidity controlled (35–40%) testing environment which needs to be preheated to 45–50°C where the testing subject is examined in a supine position on a testing table [1]. An indicator dye, which shows a pH change with a color change, is scattered on the complete ventral skin surface (omitting the eye, ears and perioral region) (17, 70). Environmental parameters may be adapted to achieve the optimal skin temperature of 38.5–39.5°C to produce sweating in a controlled and comparable fashion. Digital images of sweating patterns are then taken and the anhydrotic skin area is divided by the total skin area and multiplied with 100. Neurological disorders may show distinct sweat patterns that deviate from the physiological sweating pattern of the entire ventral body surface. If viewed in conjunction with techniques of postganglionic sudomotor function assessment, the TST can help discriminate preganglionic from postganglionic lesions (17, 19). Probably the most significant advantage of the technique in the clinical setting is its capability to define sweat patterns topographically which may lead the way for diagnosis of neurological disorders, such as neuropathies, ganglionopathies or generalized autonomic failure. The most important limitation of TST are its high technical demands. To date, fully equipped TST chambers are available only in a few specialized autonomic laboratories centers.

### Quantitative Sudomotor Axon Reflex Sweat Test (QSART)

**Figure 1:**
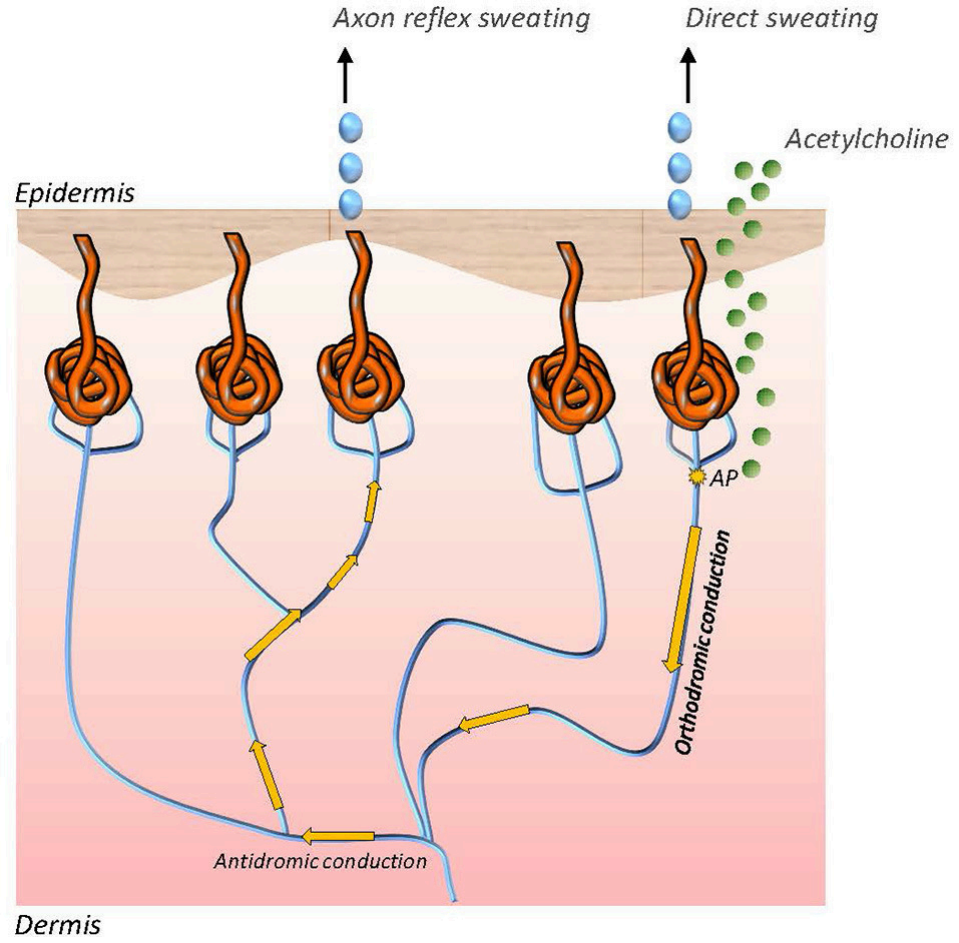
Direct and axon reflex mediated sweating in sudomotor nerve fibers. While direct sweating occurs in the akin area of iontophoretic application of acetylcholine, indirect sweating is evoked by an axon reflex in adjacent sweat glands. The axon reflex response can be assessed to study functional integrity of the sympathetic C fiber which mediates the reflex.

The QSART was first introduced in 1983 by Phillip Low and colleagues and has become the most established test of postganglionic sudomotor function (71). The technique assesses responses to pharmacological stimulation of the cutaneous axon reflex in sudomotor nerve fibers by iontophoresis of acetylcholine, a cholinergic neurotransmitter. Acetylcholine then binds to nicotinic and muscarinic receptors. Upon activation of these receptors local sweat production is evoked in the skin area where acetylcholine has been applied. This response is also referred to as the direct sweat response. The sweat response is however not restricted to direct sweating. In addition to the immediate sweat response an action potential is generated in the stimulated sudomotor nerve fibers, which is then antidromically conducted to an axon branch point to switch to adjacent sudomotor nerve fibers and the orthodromically travel to a neighboring population of sweat glands. There an indirect, axon reflex mediated sweat reaction is evoked in a skin area surrounding the area of acetylcholine iontophoresis (17, 71) (Figure 1). Quantitative evaluation of sweating in the indirect skin area is a surrogate marker of functional integrity of the sympathetic C fiber mediating the axon reflex. Local sweat output is determined as change of relative humidity over time with assessment of latency, magnitude and duration of the sudomotor response. The most commonly used skin testing sites are the forearm, proximal and distal leg and dorsum of the foot. In the clinical setting QSART should be performed if impairment of the postganglionic sudomotor nerve fibers is presumed. The most frequent observation in neurological disorders on QSART is an attenuation of sweat volume, e.g., in patients with length-dependent diabetic neuropathy (72). However, increased sweat responses to acetylcholine application may also be present in small fiber neuropathy, particularly in early disease stages, due to supersensitivity of C fibers following denervation. QSART can be used in conjunction with TST to differentiate preganglionic from postganglionic damage in sudomotor nerve fiber. QSART has high diagnostic value as it shows low variability on repeated measures and between subjects, however it is limited by high technical demands and necessity of a stale testing environment with temperature and humidity control (17, 73).

### Quantitative Direct and Indirect Test of Sudomotor Function (QDIRT)

The QDIRT was developed to assess postganglionic sudomotor function with temporal and spatial resolution of axon reflex mediated sweat responses in a technical setting which is less demanding than the QSART (74). The techniques utilizes repeated digital photography to capture axon reflex sweating upon iontophoresis of acetylcholine in a skin region pretreated with an indicator dye highlighting sweat droplet. Digital photographs are taken every 15 s over 7 min. The area of direct sweating is the skin region with direct contact to acetylcholine, whereas the area of axon reflex sweating is calculated as the total area of sweating minus the diameter of iontophoresis capsule (which is used to apply acetylcholine). Sweat droplets in the axon reflex region are analyzed for number, size and change in sweat area time. Although compared with QSART, QDIRT might decrease technical demands; the environmental prerequisites such as controlling for temperature humidity are still considerable limiting its clinical implication. More importantly QDIRT has been rarely used in research studies to date. Therefore, normative data to compare individual clinical diagnostic observations with are lacking. Multicentric prospective research of sudomotor (and pilomotor) function in patients with Parkinson's disease and healthy controls is currently under way (75).

### Sympathetic Skin Response (SSR)

**Figure 2:**
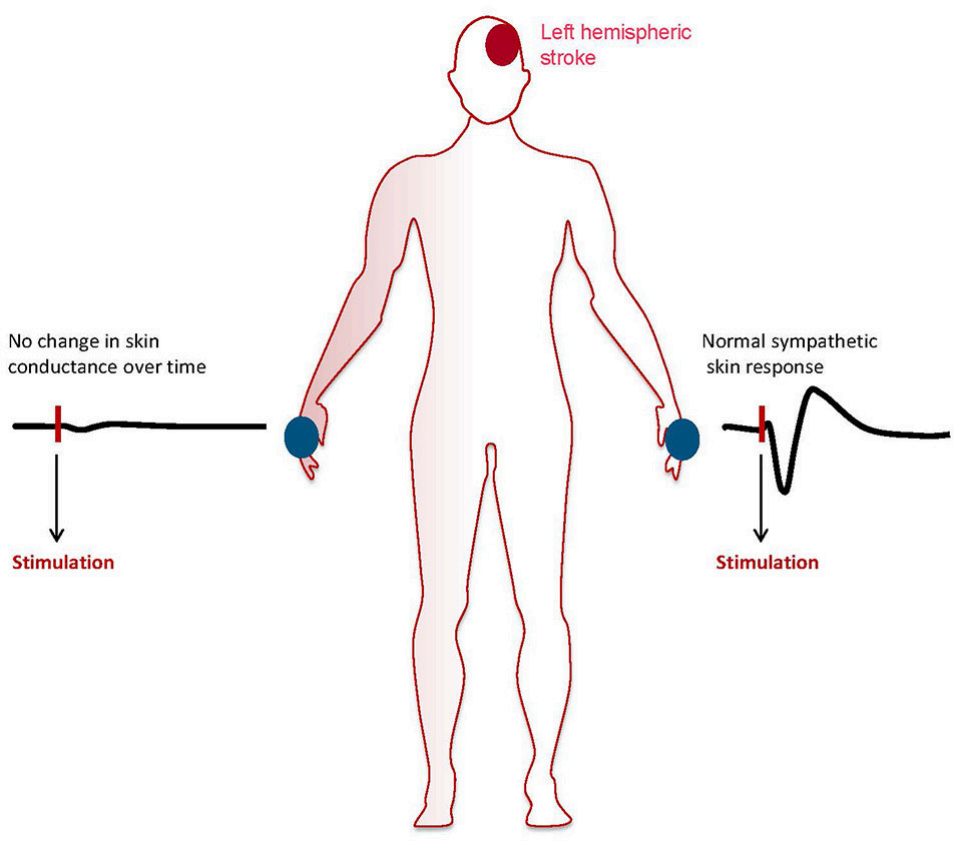
Patient with left hemispheric acute ischemic stroke showing abolished sympathetic skin response on the right hand and normal responsitivity on the left hand. The graphs show skin conductance levels (micro Sievert) over time after sympathetic stimulation.

Assessment of sympathetic skin responses with continuous measurement of electrodermal activity following sympathetic stimulation is performed with a surface electromyography electrode placed on the patient's palm or sol and a reference electrode (76, 77). Sympathetic stimulation can be undertaken by electrical stimulation or deep inspiration. Environmental factors should be well controlled for with stable light conditions and room temperature between 22 and 24°C. In addition humidity should be controlled and kept stable. Data is expressed graph indicating changes of skin conductance level over time and is analyzed for latency and amplitude following sympathetic stimulation. SSR has been investigated extensively in research studies, e.g., in patients with spinal cord trauma or diabetic neuropathy. The technique has yielded high sensitivity for changes in electrodermal conductance following emotional responses. Therefore, it is frequently used in lie detector systems as well as in psychophysiological studies (78, 79). However, due age dependent decline in sympathetic responsiveness and high interindividual variability its use in individual patients is largely limited to conditions were complete absences of the response on one testing site can be compared with recordable responses on a separate testing site (Figure 2). Although extensive research has been undertaken to define sensitivity and specificity of SSR, the mechanisms mediating this somato-sympathetic reflex are poorly understood to date (77).

### Sensitive Sweat Test (SST)

The Sensitive Sweat Test evaluates sweat secretion of each singular sweat gland and additionally captures quantity, location, and distribution of active sweat glands (77). Postganglionic sudomotor function is assessed upon iontophoresis of pilocarpine solution followed by staining of the stimulated skin region with povidone-iodine to highlight sweat droplets. A small video camera is placed on the skin are where iontophoresis is performed on.

Recording of the color change of the indicator dye due to pilocarpine induced sweating enables evaluation of sweat gland responsiveness with spatial and temporal resolution with the area of each color spot being proportional to sweat volume produced by the corresponding sweat gland and spread rate of each spot being proportional to sweat production over time. Pilocarpine is a direct cholinergic agonist, acting by activating tubular M3-receptors located on sweat glands. Axon-reflex responses are observed following pilocarpine application. The SSR therefore differs from QSART and QDIRT which primarily aim to evaluate neurogenic axon reflex mediated sweat responses. The technique is relatively fast to perform and requires a comparatively uncomplicated technical setting, highlighting its potential clinical use.

### Spoon Test

The spoon test has been designed as non-quantitative bedside screening test of sudomotor dysfunction (80). It is based on the observation that smooth sliding of the convex side of a spoon is impeded by anhidrotic dry skin. The technique shows highest specificity and sensitivity for detection of anhidrosis when performed on the chest or forehead (80, 81). Although the techniques is extremely easy to perform it lacks any quantitative analysis of sudomotor function and its results depend on the investigator's subjective perception of the smoothness the spoon slides over the skin with. However, its sensitivity as a bedside screening tool exceeds that of other available screening tools such as visual inspection of the skin surface and might therefore be a valuable addition to standard physical examination in patients suspected of having a disease which potentially affects autonomic sudomotor function (81).

### Sudoscan

Sudoscan is a recently developed technique which utilizes reverse iontophoresis and chronoamperometry to assess chloride ion concentration of the skin as a measure of sudomotor output. Applied electric current (incremental on the anode) induces a shift of chloride ions from the sweat glands to the external skin surface resulting in a current between the anode and a reference electrode which is proportional to the cutaneous chloride concentration. The technique is easy to perform and has been studied in patients with diabetic neuropathy (82–84). However, it remains to be determined whether sudoscan captures functional integrity of sweat glands, sudomotor nerve fibers or both to increase its diagnostic value in the diagnostic work up of patients with sudomotor dysfunction.

## Summary

In order to do justice to the variety of autonomic reflex systems in the autonomic functional laboratory, a combination of several different functional tests in the form of an autonomic test battery makes sense. No autonomic functional test alone is sufficiently valid. Functional tests to assess the parasympathetic nervous system (e.g., HRV in metronomic respiration) should be combined with functional tests to assess the sympathetic nervous system (e.g., blood pressure changes during orthostatic and Valsalva maneuvers).

In order to quantify the severity of an autonomic dysfunction, within a test battery such as the Ewing test battery (24) established for diabetic autonomic neuropathy, an evaluation system (0 points = physiological response, 1 = borderline response, 2 = pathological response) can be used to calculate an “autonomic test value” whose changes can be assessed, for example, in the course of a disease. Depending on the patient's symptoms or medical suspicion, more specific autonomic functional tests such as the carotid sinus massage presented here should also be carried out in special cases.

The practical implementation of autonomic functional diagnostics can be regarded as very safe. In essence, autonomic functional diagnostics can be performed without major patient stress and usually non-invasive. Nevertheless, due to possible heart rhythm disturbances, the ECG should be recorded continuously during the entire examination. In the case of an orthostatic test (change from the horizontal to the vertical body position), the subject should lie down immediately with the help of the examiner in the event of a drop in blood pressure or dizziness symptoms.

Due to the many interfering factors, autonomic functional diagnostics is relatively susceptible to external and internal influences, some of which can complicate the interpretation of test results. This makes standardized patient preparation and test execution all the more important in autonomic functional diagnostics. Due to the complexity of the autonomic nervous system, many phenomena have not yet been clarified, so that autonomic functional diagnostics is in a constant state of flux. For this reason, further training and cooperation between the various disciplines are crucial in the field of autonomic functional diagnostics.

Although less established and less widely used than cardiovascular autonomic testing, sudomotor assessment has been of increasing interest to both research studies and clinical diagnostic assessment. Improvement of precision and external validity as well as reduction of technical demands made this possible. Moreover, research has shown that neurogenic sweating is among the earliest clinical signs of a variety of autonomic neuropathies and neurodegenerative disorders highlighting diagnostic value of these techniques. However, further research is urgently needed to generate normative data sets beyond the well-studied QSART technique which shows high precision and low variability but is technically demanding. Normative data and detailed studies of sensitivity, specificity and external validity of newer techniques such as SST, QDIRT and Sudoscan are needed.

In summary, the established functional tests of the cardiovascular autonomic nervous system presented here are sufficiently standardized and well-proven. In most cases, they allow for an assessment of the cardiovascular autonomic nervous system by simple means. The daily application possibilities of these procedures in clinical routine should lead to the establishment of an autonomic functional laboratory in addition to the existing EEG, EMG, or ultrasound laboratories, which, in addition to routine diagnostics of autonomic functional disorders, can also offer a more specific diagnostics of cardiovascular autonomic functions. Although sudomotor testing using QSART and TST is also well studied, future technical improvement and research is needed to provide a set of sudomotor diagnostic techniques which yields a level of practicability and precision that is comparable to cardiovascular autonomic testing. Early and often profound affection of the sudomotor nervous system in prevalent neurological disorders such as synucleinopathies highlights the urgent need for well-designed studies in large cohorts with sudomotor dysfunction.

## Author Contributions

TZ and TS: literature analysis and design of the study and writing the manuscript.

### Conflict of Interest Statement

The authors declare that the research was conducted in the absence of any commercial or financial relationships that could be construed as a potential conflict of interest.
